# AI based prediction of chemotherapy response in cancer patients: A cross-sectional study

**DOI:** 10.6026/973206300221515

**Published:** 2026-03-31

**Authors:** Vipin Kharade, Tapesh Pounikar, Chetna Devkar

**Affiliations:** 1 Department of Radiation Oncology, All India Institute of Medical Sciences Bhopal, Madhya Pradesh, India; 2 Department of Radiation Oncology, Chhindwara Institute of Medical Sciences, Chinaware, Madhya Pradesh, India; 3 Department of Computer Sciences & Engineering, National Institute of Technology, Bhopal, Madhya Pradesh, India

**Keywords:** Artificial intelligence, chemotherapy response, machine learning, oncology, personalized medicine

## Abstract

Predicting chemotherapy response remains challenging due to the variability in tumor biology, genetics and host factors. Therefore, it is of interest to evaluate the effectiveness of AI-based predictive models in assessing chemotherapy response in cancer patients. AI models demonstrated high accuracy in distinguishing responders from non-responders, with those integrating multiple data types outperforming single-domain models. The integration of clinical, radiological and laboratory data significantly enhanced prediction accuracy. Therefore, this study advances knowledge by highlighting the potential of AI in revolutionizing chemotherapy response prediction and contributing to more targeted, effective cancer treatments.

## Background:

Cancer is one of the leading causes of illness and mortality in the world, despite significant advances in diagnostic and treatment techniques [1]. Chemotherapy remains a cornerstone of cancer treatment; nevertheless, response to chemotherapy varies significantly between patients due to tumor heterogeneity, genetic variations and host-related variables [2]. In actual practice, ineffective chemotherapy not only delays optimal treatment but also exposes patients to unnecessary toxicity and costs [3]. Traditional techniques for predicting chemotherapy response rely on clinico pathological characteristics, imaging results and molecular biomarkers, which frequently fail to provide adequate prediction accuracy when used alone [4]. The fast development of artificial intelligence (AI), particularly machine learning (ML) and deep learning techniques, has made it possible to analyze massive and complex datasets that were previously beyond human cognitive capacity [5]. AI systems can detect subtle, non-linear trends in multidimensional data, making them ideal for oncology applications with complicated interactions between factors [6]. Recent studies have shown that AI-based models can predict treatment response, survival outcomes and disease recurrence across a wide range of malignancies [7]. Radiomics, genomes and electronic health record (EHR)-based AI models have been demonstrated to predict chemotherapy sensitivity with greater accuracy than traditional statistical approaches [8]. Despite these advancements, real-world clinical use remains low due to concerns about model interpretability, generalizability and ethical considerations [9]. In the Indian healthcare setting, resource restrictions highlight the importance of efficient technologies that can guide tailored therapy and improve treatment outcomes [10]. Therefore, it is of interest to investigate the role of AI-based predictive models in assessing chemotherapy response among cancer patients in a cross-sectional environment.

## Methodology:

This hospital-based cross-sectional study was conducted in the Department of Oncology at a tertiary care teaching hospital, including cancer patients receiving chemotherapy during the study period after obtaining informed consent. Inclusion criteria consisted of histologically confirmed malignancy, patients receiving at least one cycle of chemotherapy and availability of complete clinical, laboratory and imaging data. Exclusion criteria included patients receiving only palliative care, incomplete medical records and poor- quality imaging data. Data were collected from medical records and included demographic variables, tumor type, stage, histology, laboratory parameters, radiological response (RECIST criteria) and chemotherapy regimen. Machine-learning algorithms such as logistic regression, random forest and support vector machines were used to develop AI models, with model training and validation performed using standard cross-validation techniques. Chemotherapy response was classified as responder or non-responder based on radiological and clinical assessment and model performance was evaluated using accuracy, sensitivity, specificity and ROC-AUC values.

## Results:

The study included a total of 120 cancer patients receiving chemotherapy. The mean age of patients was 52.4 ± 11.6 years, with a slight male predominance. Most patients presented with stage III or IV disease. The detailed baseline characteristics are summarized in Table 1 (see PDF). Most patients were in the 40-60 years age group, with a male predominance. Breast cancer was the most common malignancy and the majority of patients presented with advanced-stage disease (Stage III and IV). Responders constituted 61.7% of patients, while 38.3% were non-responders, indicating that a substantial proportion of patients did not achieve an adequate response to chemotherapy. Table 2 (see PDF) shows the chemotherapy response distribution. A significant proportion (61.7%) of patients was responders, while 38.3% were non-responders. Table 3 (see PDF) presents the performance metrics of the AI models. The Random Forest model demonstrated the highest accuracy (87.4%), sensitivity (85.1%), specificity (88.2%) and ROC-AUC (0.91), outperforming both the Logistic Regression and Support Vector Machine models. Among the evaluated models, the random forest algorithm demonstrated the highest accuracy and ROC-AUC, outperforming logistic regression and support vector machine models.

## Discussion:

Predicting chemotherapy response is still a significant unmet need in oncology and AI-based techniques present a viable answer by combining disparate data sources [11]. In the current investigation, AI models were very accurate in discriminating responders from non-responders, which is consistent with earlier findings. According to research, machine-learning models outperform traditional predictive methods by capturing intricate interplay between tumor biology and patient-specific characteristics [12]. Radiomics-based AI models, in particular, have proven effective at predicting chemotherapy response in breast, lung and colorectal malignancies [13]. The higher performance of multimodal models observed in this investigation is consistent with findings that combining clinical, imaging and laboratory data increases predictive power [14]. This method is consistent with real-world clinical decision-making, which takes into account various data streams at the same time. However, problems such as a lack of consistent datasets, inherent algorithmic bias and limited interpretability continue to prevent widespread clinical implementation [15]. Ethical considerations around data privacy and openness must be resolved before normal implementation. Despite their shortcomings, AI-driven prediction models have enormous potential in customized oncology. Early identification of likely non-responders allows clinicians to adapt treatment techniques, limit toxicity and enhance overall results [16].

## Conclusion:

AI-based predictive models have shown substantial promise in anticipating chemotherapy response in cancer patients. Their use into clinical oncology practice may enable more individualized treatment planning and enhance patient outcomes. Additional large-scale, multicentric researches are needed to validate these findings.
